# Environmental Factors Determining the Distribution Pattern of Chironomidae in Different Types of Freshwater Habitats

**DOI:** 10.3390/insects16050501

**Published:** 2025-05-07

**Authors:** Nataša Popović, Jelena Đuknić, Nikola Marinković, Bojana Tubić, Ana Atanacković, Djuradj Milošević, Maja Raković

**Affiliations:** 1 Department of Hydroecology and Water Protection, Institute for Biological Research “Siniša Stanković”—National Institute of the Republic of Serbia, University of Belgrade, Bulevar Despota Stefana 142, 11108 Belgrade, Serbia; jelena.djuknic@ibiss.bg.ac.rs (J.Đ.); nikola.marinkovic@ibiss.bg.ac.rs (N.M.); bojana@ibiss.bg.ac.rs (B.T.); adjordjevic@ibiss.bg.ac.rs (A.A.); rakovic.maja@ibiss.bg.ac.rs (M.R.); 2 Department of Biology and Ecology, Faculty of Sciences and Mathematics, University of Niš, Višegradska 33, 18000 Niš, Serbia; djuradj.milosevic@pmf.edu.rs

**Keywords:** aquatic insects, Chironomidae, environmental factors, indicator taxa, distribution

## Abstract

Chironomidae are characterised by cosmopolitan distribution, high abundance and diversity in different aquatic environments, which makes them ideal for studying changes in freshwater ecosystems. To understand the environmental factors influencing chironomid communities, we analysed how altitude and waterbody type (hydromorphological features) affect their composition at 75 study sites from 49 watercourses. A total of 110 chironomid taxa from five subfamilies were identified, with *Prodiamesa olivacea*, *Rheocricotopus fuscipes* and *Cricotopus bicinctus* being the most frequent species. The lowest values of all alpha diversity components were recorded in communities collected from watercourses at altitudes up to 500 m a.s.l., while the highest values were observed in small mountainous rivers and streams. Beta diversity showed that taxa turnover was the dominant component in all situations analysed. Communities in large rivers with fine substrate were characterised by the lowest taxa turnover and the highest levels of nestedness, indicating the existence of an ecological gradient that reduces the number of taxa from one site to another. We identified indicator taxa for different altitudes, as well as groups of taxa that are typical for different waterbody types. Furthermore, the combination of four water parameters (oxygen saturation, conductivity, concentration of ammonium and nitrates) had the strongest influence on the chironomid community composition in the studied watercourses.

## Simple Summary

Chironomidae (non-biting midges) are globally distributed aquatic insects that serve as bioindicators of the state of freshwater ecosystems due to their diverse ecological niches and responsiveness to environmental changes. A study of 75 study sites showed how altitude and hydromorphological features shape their communities. These results emphasise the utility of Chironomidae in tracking changes in freshwater ecosystems, particularly through their responses to altitude-dependent climatic variables and waterbody-specific physicochemical conditions.

## 1. Introduction

Freshwater ecosystems are very vulnerable and as such they are the most exposed to global changes, even more than terrestrial or marine [1,2]. Environmental change has a strong impact on aquatic organisms. The structure of macroinvertebrate communities is influenced by many natural and anthropogenic stressors, such as hydromorphological and hydrogeological features, the physical and the chemical contaminants of the water and sediment, as well as the substrate type and interactions with other organisms [3,4,5]. An important part of macroinvertebrate communities in various aquatic environments are larval stages of Chironomidae (Diptera, Nematocera), since they could be abundant and diverse. They have cosmopolitan distribution, colonise a wide range of habitats, including those with unfavourable conditions such as water scarcity, low pH, low oxygen levels or high pollution [6]. Due to their wide distribution and diverse environmental preferences, chironomids are ideal for studying changes in freshwater ecosystems [7]. Despite their high indicator potential, chironomids are often overlooked in ecological studies due to relatively complex taxonomy and identification challenges. It is therefore important to expand our knowledge of this family, in order to understand how environmental stressors affect these communities [8,9]. Furthermore, knowledge of these relations is necessary for modelling and predicting the distribution of species that may change in response to an environmental change, as well as for conservation efforts [10,11]. As most of habitats are altered by human activities, and the climate continues to warm, data from the study of ecological patterns can help develop strategies to protect biodiversity and maintain ecosystem services [12].

To better understand the environmental factors influencing the diversity and distribution of this important group of aquatic macroinvertebrates, here we analyse how altitudinal and environmental gradients shape the composition of the chironomid communities in Serbia. Serbia is a country located in the central part of the Balkan Peninsula, partly covering the southern part of the Pannonian Plain. It is one of the most complex regions in Europe, characterised by rich and specific ecosystems and great biodiversity. The reasons for this are the heterogeneous environmental conditions, the geographical location, the complex relief at the position were continental and Mediterranean influences interface [13]. The environmental factors also contribute significantly to the uniqueness of the aquatic ecosystems. Furthermore, Serbia is considered as a “diversity hotspot” since it was a glacial refugia during the last ice age [14].

The main goals of this study were: (1) to analyse the diversity and composition of Chironomidae communities; (2) to analyse influence of altitudinal gradient and waterbody type on their distribution; (3) to determine whether there are taxa in the analysed community that are specific for a particular altitude or waterbody type; and (4) to determine their response to particular and/or group of various environmental predictors in various freshwater habitat types in Serbia.

## 2. Materials and Methods

### 2.1. Study Area

Serbia is a country located in the central part of the Balkan Peninsula. According to macroinvertebrate communities and general natural and biogeographical features, the territory of Serbia is a part of five ecoregions: ecoregion 5—Dinaric Western Balkan, ecoregion 6—Hellenic Western Balkan, ecoregion 7—Eastern Balkan, ecoregion 10—Carpathian, and ecoregion 11—Pannonian Lowland [15]. There are three climate types with several subtypes in Serbia. According to the Köppen climate classification, most parts of Serbia have a moderately warm and humid climate (Cfb and Cfa). The continental climate type with humid continental mild summer, wet all year (Dfb) is present on the lowland terrains of Central Serbia. At higher altitudes, the climate is moderately cold and humid, while only the parts with the highest mountains have a cold and humid climate (Dfc). Detailed descriptions of the climate components of Serbian territory were given by Milovanović et al. [16,17]. The highest average annual air temperature is in the capital of Serbia (Belgrade), where it exceeds 12 °C. On the highest peaks of the mountains in the southwest and southeast of the country, the average annual air temperature is below 2 °C. Precipitation varies from 550 to 600 mm (mainly in the northern part of the country) to over 1100 mm on the highest mountains in the south-west [17].

### 2.2. Field and Laboratory Work

**Figure 1:**
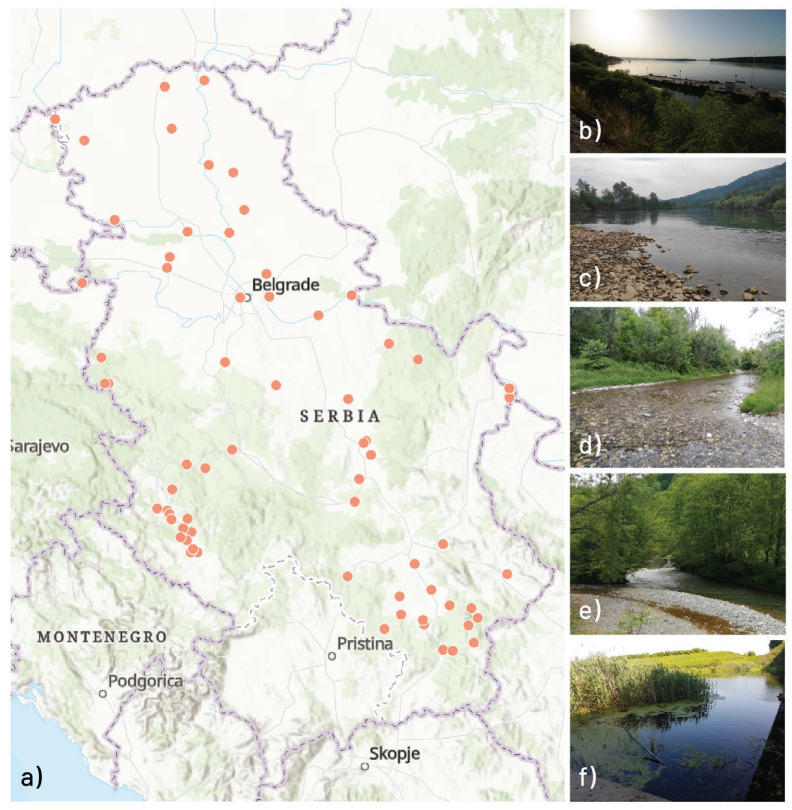
Study area with (**a**) the geographic position of 75 study sites across Serbia, and photo examples of study sites (**b**) WBT 1—large rivers with fine substrate (**c**) WBT 2—mix of large and medium rivers with coarse substrate (**d**) WBT 3—small watercourses with coarse substrate (**e**) WBT 4—small mountainous rivers and streams (**f**) WBT 5—slow flowing/stagnant waters (artificial canals and reservoirs).

A study was carried out in spring 2019 and summer 2020, and it included 75 study sites from 49 watercourses across Serbia (Figure 1).

In order to obtain large set of different environmental conditions, we included a variety of waterbodies: from mountain streams, upper river stretches, and downstream to the large lowland rivers as well as reservoirs and canals.

Macroinvertebrate samples were collected using a FBA hand net (mesh size 500 µm and 250 µm) applying a combination of the kick and sweep and multihabitat sampling techniques according to European Standards (EN 27828:1994) [18]. The collected material was preserved in the field with ethyl alcohol (70%), and transported to the laboratory for further analyses. Chironomidae larvae were identified to the lowest possible taxonomic level (genus, species or species groups and aggregates) using relevant identification keys [19,20,21,22,23,24,25,26,27].

During the sampling survey at each study site, following environmental parameters were also measured: water temperature (°C), electrical conductivity (µS/cm), pH, oxygen concentration (mg/L O_2_) and oxygen saturation (O_2_%) using the Horiba W-23XD multiparametric probe (HORIBA Instruments Corporation, Irvine, CA, USA). Current velocity was scored in the field by investigators and was given one of three values: 1—slow, 2—medium, 3—fast. In addition, water samples from each study site were collected and adequately transported to the laboratory, where concentrations of nitrates (NO_3_^−^) (mg/L N) (EPA 300.1) and ammonium ions (NH_4_^+^) (mg/L N) (PRI P-V 2A) were measured.

### 2.3. Data Analyses

The qualitative composition of the Chironomid communities was determined for each site, together with the frequency of taxa occurrence (F = 0–1) and their relative abundance (percentage participation).

As the environmental factors (current velocity, bottom properties, temperature, oxygen concentration) change predictably depending on the waterbody type and with increasing altitude [28], the Chironomidae communities were analysed according to waterbody type and altitudinal gradient. To define waterbody type we classified each study site according to its characteristics (hydromorphological properties) into the following groups: WBT 1—large rivers with fine substrate (silt, clay mud, and sand); WBT 2—mix of large and medium rivers with coarse substrate (gravel, stones, and rocks); WBT 3—small watercourses with coarse substrate (gravel, stones, and rocks); WBT 4—small mountainous rivers and streams and WBT 5—slow flowing/stagnant waters (artificial canals and reservoirs). The altitude gradient was also defined into three groups: ALT 1—study sites up to 500 m a.s.l.; ALT 2—study sites from 500 to 1000 m a.s.l.; ALT 3—study sites above 1000 m a.s.l. The list of study sites and their characteristics (altitude gradient and waterbody type to which the study sites belong) is shown in Table S1.

Within each above-mentioned group of the Chironomidae communities, the components of alpha diversity were analysed using the taxa richness, the Shannon entropy, the Equitability index (evenness of abundance) and the community richness index (using the taxa richness estimator, Chao-1 index). Total beta diversity was defined by two additive components: the taxa turnover, which is independent of the difference in taxa richness, and the variation in taxa composition resulting from nestedness [29]. Beta diversity was calculated for the different environmental categories (waterbody type and altitude). The FLORA software version 2013 [30] was used to analyse the components of alpha and beta diversity. Furthermore, in order to group chironomid communities among different watercourses (belonging to different altitude categories and waterbody types) cluster analysis based on Ward’s method [31] was used in FLORA software version 2013 [30]. The number of shared taxa among study sites groups was visualised in a Venn diagram using the web tool available at http://bioinformatics.psb.ugent.be/webtools/Venn/ (accessed on 15 January 2025).

To identify the taxa typical for different altitudes and waterbody types, the indicator species analysis [32] was conducted in ‘R’ (R 4.3.2 software).

The similarities in the composition of Chironomidae communities between different altitudes and waterbody types were analysed using non–metric multidimensional scaling analysis (NMDS) based on the Bray–Curtis similarity matrix. Prior to analysis, taxa abundance data were square root transformed. Two–way PERMANOVA with 999 permutations was used to test significance of differences between groups of communities in both cases, based on the Bray–Curtis similarity matrix. Canonical analysis of principal coordinates (CAP) was used to detect spatial differences in Chironomidae communities based on biological and environmental data. PERMANOVA was used to test significance of differences between/among sample grouping based on the Bray–Curtis similarity matrix, with 999 permutations. Taxa abundance data were square root transformed. These analyses were performed in PRIMER 6 v. 6.1.16 and PERMANOVA+ Version 1.0.6.

In addition, a biological environmental gradient analysis (BIO-ENV) [33] was performed to test the relationship between the communities and the environmental parameters. A Euclidean distance matrix for environmental parameters was created as the input matrix for the BIO-ENV analysis [33]. Using this matrix, BIO-ENV compares the environmental parameters with the communities’ composition, which were represented as Bray–Curtis similarity matrices. As a result of this method, which uses the Spearman rank correlation test, a subset of environmental variables that are strongly correlated with the similarity matrix of the biota is identified using PRIMER 6 v. 6.1.16 and PERMANOVA+ Version 1.0.6.

## 3. Results

In total, 110 chironomid taxa (Table S2) from five subfamilies (Tanypodinae, Diamesinae, Prodiamesinae, Orthocladiinae, Chironominae) were identified in the studied habitats. The highest number of taxa (23) was found in Marića Reka River, followed by Uvac and Zlošnica rivers with 20 taxa each, while the lowest number of taxa with only one chironomid taxon was recorded in Borkovac and Kudoš reservoirs.

The most frequently recorded species were *Prodiamesa olivacea* (Meigen 1818), *Rheocricotopus fuscipes* (Kieffer 1909), *Cricotopus bicinctus* (Meigen 1818), as well as taxa from genus *Procladius* and *Tanytarsus*, while 29 taxa were found to be site specific (Table S2). The highest abundance of chironomid taxa was found in small watercourses with coarse substrate—WBT 3 (57% of total abundance) and small mountainous rivers and streams—WBT 4 (27.50%), while it was lower in large rivers—WBT 1 and WBT 2 (11.15%) and in slow flowing/stagnant waters—WBT 5 (4.35%).

Taxa from the subfamily Chironominae were most abundant in lowland rivers—WBT 1 (55%), with *Polypedilum* gr. *scalenum* and *Chironomus* taxa dominating. The subfamily Orthocladiinae was most abundant in small watercourses with coarse substrate and in small mountainous rivers and streams (36.7% and 49%, respectively), with domination of *Rheocricotopus fuscipes* and *Tvetenia calvescens* agg. as well as taxa from the genus *Cricotopus* and *Thienemanniella*.

**Figure 2:**
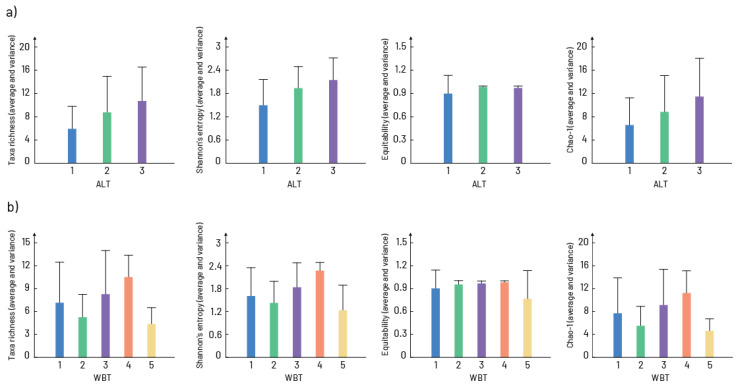
Alpha diversity and its components in relation to (**a**) altitude gradient (ALT: 1—study sites up to 500 m a.s.l.; 2—study sites from 500 to 1000 m a.s.l.; 3—study sites above 1000 m a.s.l.) and (**b**) waterbody types (WBT: 1—large rivers with fine substrate (silt, clay mud, and sand); 2—mix of large and medium rivers with coarse substrate (gravel, stones, and rocks); 3—small watercourses with coarse substrate; 4—small mountainous rivers and streams and 5—slow flowing/stagnant waters (artificial canals and reservoirs)). Bars represent average values while lines denote the variance in each group of communities.

Chironomidae communities differed significantly in relation to altitude (Figure 2a). All alpha diversity components (Shannon entropy, taxa richness and Chao 1) showed similar trends. Thus, communities recorded at study sites up to 500 m showed the lowest values, while those at the highest altitudes showed the highest values of all alpha diversity components. In relation to waterbody types (Figure 2b), the highest value of taxa richness, Shannon entropy and Chao 1 were observed in small mountainous rivers and streams (WBT4). However, the Equitability index was almost identical for communities in all types of waterbodies.

**Figure 3:**
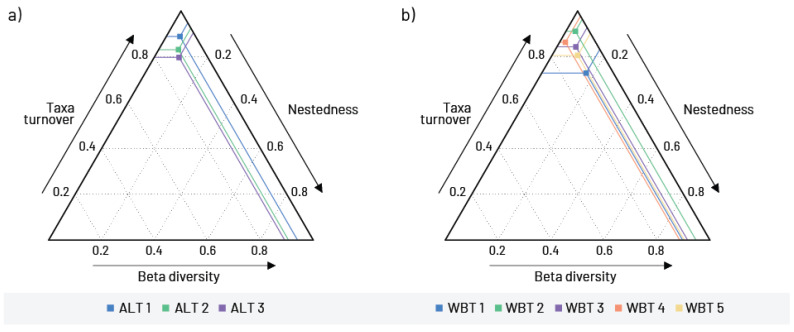
Beta diversity and its components in relation to (**a**) altitude gradient (ALT 1—study sites up to 500 m a.s.l.; ALT 2—study sites from 500 to 1000 m a.s.l.; ALT 3—study sites above 1000 m a.s.l.) and (**b**) waterbody types (WBT 1—large rivers with fine substrate (silt, clay mud, and sand); WBT 2—mix of large and medium rivers with coarse substrate (gravel, stones, and rocks); WBT 3—small watercourses with coarse substrate; WBT 4—small mountainous rivers and streams and WBT 5—slow flowing/stagnant waters (artificial canals and reservoirs)).

Beta diversity as an indicator of the diversity of conditions prevailing in different habitats, in this case at different altitudes, showed similar values (Figure 3a). Taxa turnover was the dominant component of beta diversity. It was higher than the nestedness at all altitudinal categories. However, the highest value for taxa turnover and the lowest for nestedness were found in the study sites up to 500 m altitude.

Taxa turnover was also a dominant component of beta diversity in different waterbody types. In this case, the lowest taxa turnover and the highest nestedness were found in large lowland rivers with fine substrate (Figure 3b).

**Figure 4:**
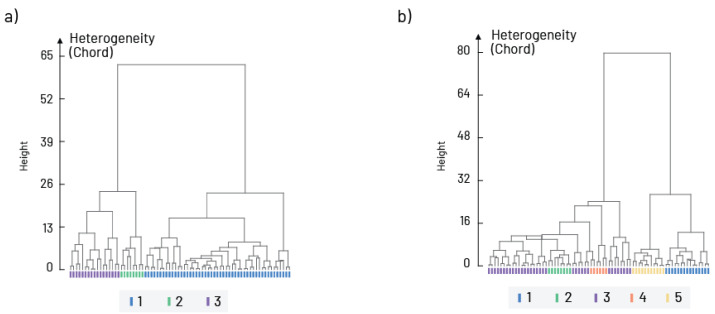
Hierarchical cluster analyses of the Chironomidae communities in relation to (**a**) altitude gradients (1—study sites up to 500 m a.s.l.; 2—study sites from 500 to 1000 m a.s.l.; 3—study sites above 1000 m a.s.l.) and (**b**) waterbody type (1—large rivers with fine substrate (silt, clay mud, and sand); 2—mix of large and medium rivers with coarse substrate (gravel, stones, and rocks); 3—small watercourses with coarse substrate; 4—small mountainous rivers and streams and 5—slow flowing/stagnant waters (artificial canals and reservoirs)).

The cluster analyses based on the similarity of taxa composition with respect to altitude and waterbody types divided the communities into two main clusters. According to altitude, the first cluster included all communities found above 500 m a.s.l., while the second cluster included only sites up to 500 m a.s.l. (Figure 4a). By waterbody type, the first cluster included communities from all rivers with hard bottom substrate, and the second cluster from large rivers with fine substrate and slow-flowing and stagnant water (Figure 4b).

**Figure 5:**
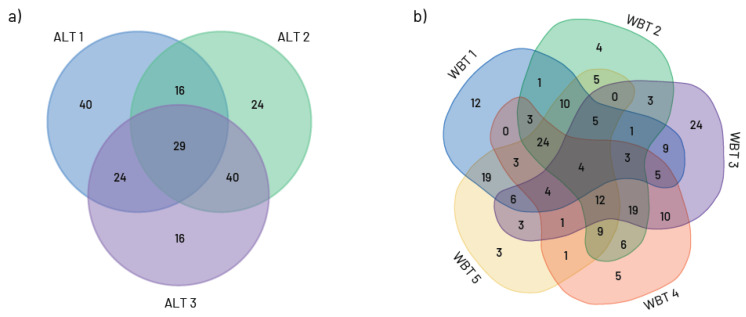
Number of taxa recorded in each study sites groups and the number of taxa shared among two or more groups of sites (**a**) altitudinal gradients (ALT 1—study sites up to 500 m a.s.l.; ALT 2—study sites from 500 to 1000 m a.s.l.; ALT 3—study sites above 1000 m a.s.l.) and (**b**) waterbody type (WBT 1—large rivers with fine substrate (silt, clay mud, and sand); WBT 2—mix of large and medium rivers with coarse substrate (gravel, stones, and rocks); WBT 3—small watercourses with coarse substrate; WBT 4—small mountainous rivers and streams and WBT 5—slow flowing/stagnant waters (artificial canals and reservoirs)).

The number of taxa that are common or exclusive to different groups of communities is represented by the Venn diagram (Figure 5). Thus, 29 of 110 Chironomidae taxa occurred in all altitudinal classes (Figure 5a), while only four taxa were common to all five analysed waterbody types (*R. fuscipes*, *C. bicinctus*, *Procladius* sp. and *Tanytarsus* sp.) (Figure 5b).

In addition, the indicator species analyses found four significant indicator species *Macropelopia nebulosi* (Meigen, 1804), *Odontomesa fulva* (Kieffer, 1919), *Rheocricotopus effusus* (Walker, 1856) and *Paratanytarsus dissimilis* (Johannsen, 1905) for sites at altitudes between 500 and 1000 m, while species *Prodiamesa olivacea*, *Paratrissocladius excerptus* (Walker, 1856), *Paracladius conversus* (Walker, 1856) were indicators for all sites above 500 m (ALT 2 and ALT 3) (Table 1).

Furthermore, indicator species analysis singled out seven significant indicator taxa for large rivers with fine substrate (WBT 1): *Polypedilum scalaenum* Schrank, 1803, *Microchironomus tener* (Kieffer, 1918), *Dicrotendipes nervosus*, *Chironomus acutiventris* Wuelker and Ryser 1983, *Parachironomus* gr. *gracilior*, *Paralauterborniella nigrohalteralis* (Malloch, 1915) and *Harnishia fuscimanus* Kieffer, 1921 (Table 2). In small mountainous rivers and streams (WBT 4) *Tvetenia clavescens* (Edwards, 1929), *Rheotanytarsus* sp., *Micropsectra bidentata*, *Odontomesa fulva*, *Thienemanniella* sp., *Rheocricotopus effusus* and *Rheocricotopus chalybeatus* were singled out as indicator taxa, while *Parachironomus* gr. *gracilior*, *Ablabesmyia longistyla* Fittkau, 1962 and *Polypedilum nubifer* Skuse, 1889 were indicators for WBT 5—artificial canals and reservoirs (Table 2).

**Figure 6:**
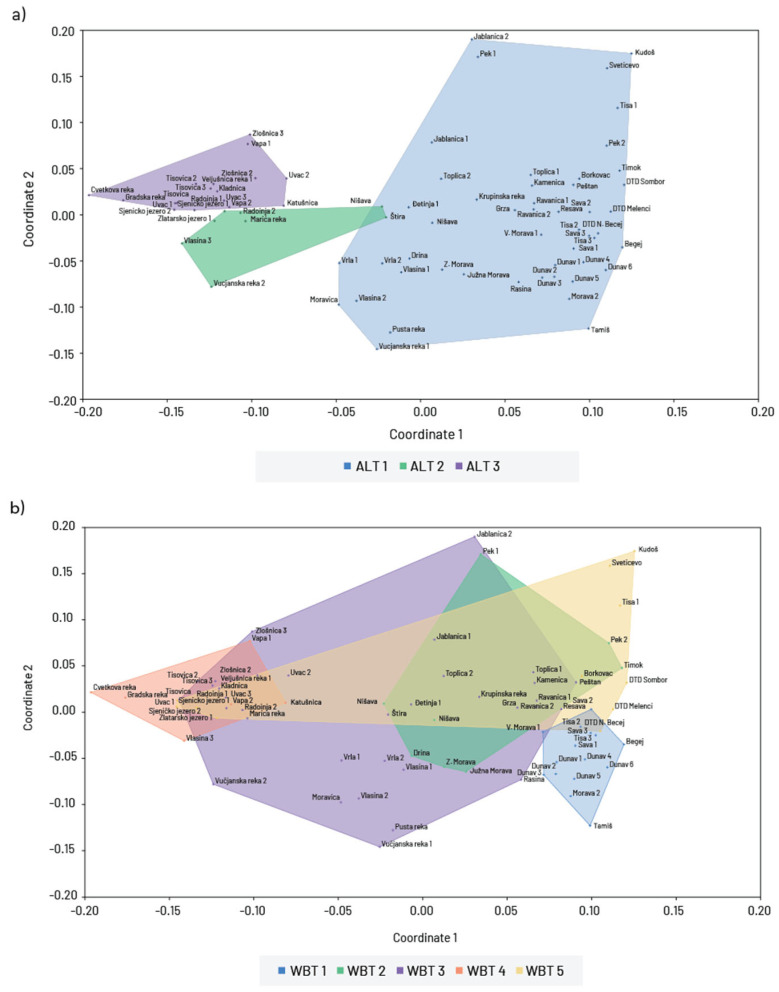
Non-metric multidimensional scaling (NMDS) of chironomid communities from different (**a**) altitude gradients (ALT 1—study sites up to 500 m a.s.l.; ALT 2—study sites from 500 to 1000 m a.s.l.; ALT 3—study sites above 1000 m a.s.l.) and (**b**) waterbody types (WBT 1—large rivers with fine substrate (silt, clay mud, and sand); WBT 2—mix of large and medium rivers with coarse substrate (gravel, stones, and rocks); WBT 3—small watercourses with coarse substrate; WBT 4—small mountainous rivers and streams and WBT 5—slow flowing/stagnant waters (artificial canals and reservoirs)) based on the Bray–Curtis similarity matrix.

According to chironomid composition, sites were grouped into three clearly distinct clusters, which correspond well with three predefined groups based on the altitude (Figure 6a). There was no clear pattern in similarity of communities observed trough the waterbody type groups (Figure 6b).

The results of the two-way PERMANOVA (Table 3) showed statistically significant differences between the groups of communities in both cases the waterbody type and altitude significantly influence the composition of the communities. The interaction between waterbody type and altitude is also significant. Waterbody type has the strongest effect (SS 27,437), followed by altitude and their interaction.

**Figure 7:**
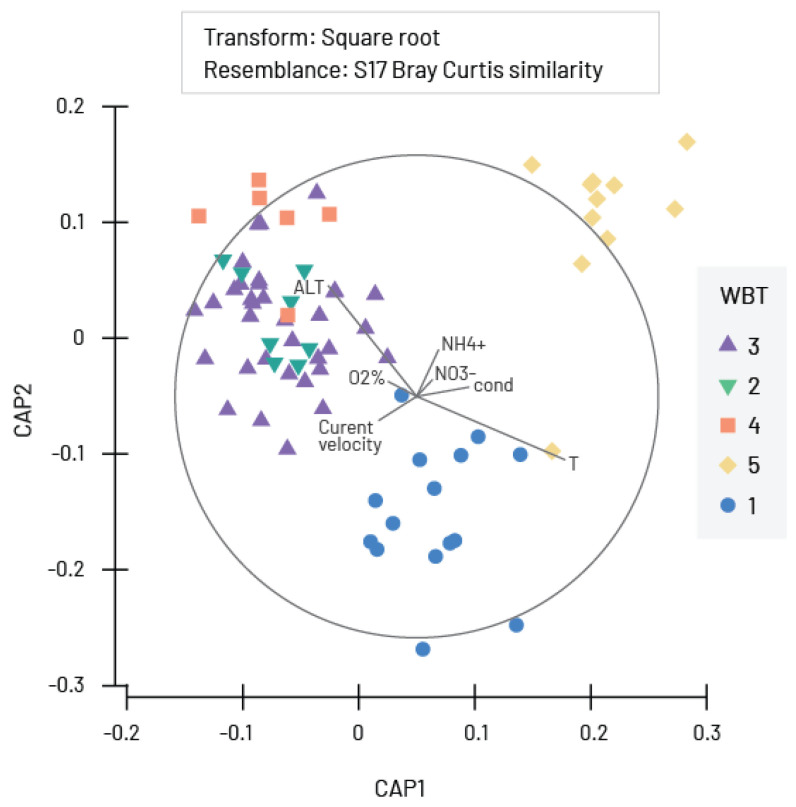
Canonical analysis of principal coordinates (CAP) of chironomid samples collected throughout Serbia and correlations with environmental parameters WBT 1—large rivers with fine substrate (silt, clay mud, and sand); WBT 2—mix of large and medium rivers with coarse substrate (gravel, stones, and rocks); WBT 3—small watercourses with coarse substrate; WBT 4—small mountainous rivers and streams and WBT 5—slow flowing/stagnant waters (artificial canals and reservoirs; cond—electrical conductivity; O_2_%—oxygen saturation; NO_3_^−^—nitrates; NH_4_^+^—ammonium ions; ALT—altitude.

Even though NMDS did not show large overlap of polygons representing communities grouped by type of waterbody, Canonical analysis of the principal coordinates (CAP) showed that the Chironomidae communities were divided into three groups according to the WBT (Figure 7). The first group represents communities in rivers with hard substrate (WBT 2, 3 and 4), where mutual overlap was observed, while the other two groups WBT 5 (communities in slow-flowing and stagnant waters) and WBT 1 (large rivers with fine substrate) show no mutual overlap. PERMANOVA revealed high differences (F = 7.183, *p* = 0.0001) among these three groups and pairwise comparison showed evidence of differences between groups (Table 4). The communities in large rivers with fine substrate were correlated with water temperature, while the communities in stagnant waters were influenced by nutrients (nitrogen compounds). The communities in watercourses with hard substrate (WBT 2, 3 and 4) were all positively correlated with altitude and oxygen saturation in water (Figure 7).

Finally, using BIO-ENV analysis, we found combinations of environmental parameters that significantly (*p* = 0.01) determine the chironomid community composition (Table 5). The first model provided the combination of four parameters oxygen saturation, conductivity, concentration of ammonium ions and nitrates that influenced the spatial variability of the chironomid communities (Spearman’s rank correlation coefficient, Rho = 0.224; *p* = 0.01).

## 4. Discussion

The territory of Serbia is characterised by rich and specific ecosystems with great biodiversity [13], which is also reflected in the diverse Chironomidae fauna. The identification of 110 chironomid taxa from five subfamilies (Tanypodinae, Diamesinae, Prodiamesinae, Orthocladiinae, Chironominae) in the waterbodies across Serbian territory confirmed that the chironomid diversity and their distribution was consistent with the data from neighbouring countries (Bulgaria, Hungary and Croatia) [34,35,36]. As a significantly higher diversity was confirmed in Romania [37], it is expected that the number of taxa of this dipteran group could also be higher in the above-mentioned countries, depending on the type of ecosystem studies and the intensity of research (seasonality and frequency of sampling) [36]. The subfamily Chironominae proved to be the most diverse and abundant subfamily, followed by the subfamily Orthocladiinae, which was also reported in other studies [7,36,38]. In correspondence with the literature data, the abundance of Chironominae (especially Chironomini) is increasing in lowland waters with fine sediments [7,39]. On the other hand, the most frequently found taxa in our study were from the subfamilies Orthocladiinae and Prodiamesinae, which are commonly found in mountainous regions where they are relatively diverse [40,41]. Geographical location of the studied areas and the waterbody type (hydromorphological features) explain most of the overall variation among chironomid communities. Furthermore, the community composition is influenced by the local climate and site-specific environmental conditions, which can vary at different altitudes [42]. The communities that we found above 500 m a.s.l. and those at lower altitudes (below 500 m a.s.l) undoubtedly differed from one another. This can be a direct or indirect consequence of elevation-dependent biotic and abiotic factors. Substrate type and current velocity affect food availability, vegetation, and microhabitat diversity [43]. Thus, the richness and abundance of taxa from the genera *Chironomus*, *Tanytarsus*, *Rheotanytarsus*, *Polypedilum*, *Cricotopus* and *Procladius* increase in lowland rivers with fine sediment where water temperature increases, which reduces the availability of dissolved oxygen, similar to findings of Villamarín et al. [42]. The highest alpha diversity was observed at higher altitudes above 1000 m a.s.l.with hard bottom substrate, which corroborates the results of a study conducted in Northern Italy [44]. On the other hand, other authors found that highest alpha diversity is between 600 and 800 m a.s.l. [45]. The most frequently recorded and abundant species at altitudes above 500 m was found to be *Prodiamesa olivacea*. This cosmopolitan species is widespread throughout most of European freshwaters [46], preferring high altitudes [47]. Although some authors are of the opinion that altitude does not affect the distribution of chironomid species [48], our findings support numerous studies that showed that species distribution is correlated with altitude as well as longitudinal zonation [9,42]. Altitude-related factors such as temperature, substrate type and nutrient content interact with current velocity and oxygen and create different environmental conditions in mountainous and lowland streams. These conditions influence the composition and abundance of chironomid communities by selecting species that are adapted to specific combinations of these altitude-dependent factors [42].

However, altitude not only was responsible for the differences in chironomid community composition, but also the hydromorphological characteristics that define different types of waterbodies. Other studies investigating river typologies also distinguish groups of Chironomidae communities based on river types [49,50,51]. We found groups of indicator species that were specific to the waterbody type/altitude (Table 1 and Table 2) that were consistent with literature data [20,23,48], confirming their good potential as typology predictors. While *Ablabesmyia longistyla* is described as a common species in running waters, in our study area it was more abundant in artificial canals and reservoirs which is consistent with its findings in the Netherlands, where it is more common in stagnant waters [20].

Chironomid diversity observed through alpha and beta diversity components was high. Although the alpha diversity of the chironomid communities in large and medium rivers with coarse substrate (WBT 2) was lower, suggesting low taxa richness of individual habitats, high beta diversity indicates presence of pronounced diversity between these habitats. High values of the beta diversity components (taxa turnover) in a particular habitat type mean that the replacement of taxa is high, and the fauna is more heterogeneous. The chironomid communities in large rivers with fine substrate (WBT 1) were also characterised by higher nestedness (component of beta diversity), indicating the existence of an ecological gradient that reduces the number of taxa from one site to another. The stagnant waters sites as well as those along large rivers showed a decrease in alpha diversity, a lower chironomid taxa richness and the development of denser populations of *Chironomus* taxa. These sites were exposed to greater anthropogenic pressure as they are close to urban areas, industry and farmland. Positive correlation between chironomid communities in stagnant waters and nutrients such as nitrogen compounds, as well as conductivity, was revealed which is consistent with the knowledge that tolerant taxa prevail at sites exposed to strong anthropogenic influence [52,53]. Other parameters such as water temperature, current velocity, bottom substrate, dissolved oxygen, oxygen saturation, and hydro-morphology also explain variation among chironomid communities [4,54,55,56,57,58,59]. Thus, communities at higher altitude inhabiting rivers and streams with hard rock bottoms (WBT 2, 3, 4) were characterised by taxa preferring oxygen-rich habitats and high current velocity, like Orthocladiinae subfamily [40,60]. The literature data consider other important factors influencing the distribution of species. For instance, Syrovátka et al. [43] note substrate type as one of the most important factors, while habitat heterogeneity is considered as one of the crucial factors for the presence and distribution of chironomids and other aquatic invertebrates in various studies [61,62,63]. According to Lindegaard [64], temperature is an important factor influencing the distribution of Diamesinae and the cold-stenothermic Orthocladiinae, while other factors such as habitat type and water chemistry, physical conditions and substrate characteristics have a greater influence on the distribution of Chironomini [65]. Diamesinae are considered dominant in the Alps and other high mountain regions [50,66,67,68,69,70,71], while in our study they were present in various waterbodies but neither abundant nor diverse (only three taxa). Upon observing all the results, it can be concluded that the distribution of chironomid fauna of the studied area is shaped by different drivers such as altitude and waterbody type along with covarying environmental parameters which change predictably across these gradients.

## 5. Conclusions

Diversity and community composition of Chironomidae communities on the territory of Serbia were rich and comparable to those in the neighbouring countries. Altitude and hydromorphological characteristics of a waterbody were the key drivers for distribution of taxa, and some of the taxa have shown to be reliable typology predictors. In spite of the fact that the communities in the different waterbody types appeared to be homogeneous at first sight, those in stagnant waters (reservoirs and canals) and large lowland rivers with fine substrate differed significantly in their composition from the others. Nitrogen compounds, oxygen concentration, and current velocity were also important factors that influenced studied communities. In addition, we found a strong influence of altitude in shaping chironomid communities. These results are important for understanding the ecological drivers of species distribution and diversity. Bearing in mind that Chironomidae inhabit various habitats, understanding their community composition and species distribution as well as the ability to predict how these communities will respond to environmental change is necessary for the habitat and ecosystem conservation measures.

## Figures and Tables

**Table 1:** Indicator values for Chironomidae taxa from different altitudes. Only taxa with significant values were shown (Significance codes: 0.001 ‘**’ 0.01 ‘*’); ALT 1—study sites up to 500 m a.s.l.; ALT 2—study sites from 500 to 1000 m a.s.l.; ALT 3—study sites above 1000 m a.s.l.

ALT 1	A	B	Stat	*p* Value
*Macropelopia nebulosa*	1.0000	0.2500	0.500	0.011 *
*Odontomesa fulva*	0.9199	0.2500	0.480	0.047 *
*Rheocricotopus effusus*	0.8947	0.2500	0.473	0.023 *
*Paratanytarsus dissimilis*	0.4762	0.3750	0.423	0.033 *
ALT 3				
*Tanytarsus* spp.	0.8710	0.7647	0.816	0.004 **
*Conchapelopia* agg.	0.8775	0.5294	0.682	0.010 **
*Tvetenia calvescens* agg.	0.9813	0.4706	0.680	0.003 **
*Microtendipes pedellus* agg.	0.9563	0.3529	0.581	0.015 *
*Corynoneura lobata*	0.9890	0.2941	0.539	0.010 **
*Cladotanytarsus* sp.	0.7862	0.3529	0.527	0.044 *
*Apsectrotanypus trifascipennis*	1.0000	0.1765	0.420	0.038 *
*Dicrotendipes notatus*	1.0000	0.1765	0.420	0.029 *
*Xenopelopia* sp.	1.0000	0.1765	0.420	0.040 *
ALT 2 and 3				
*Orthocladius* sp.	0.9785	0.4800	0.685	0.015 *
*Prodiamesa olivacea*	0.9746	0.4400	0.655	0.008 **
*Paratrissocladius excerptus*	0.9992	0.2400	0.490	0.028 *
*Paracladius conversus*	0.9944	0.2400	0.489	0.028 *

**Table 2:** Indicator values for Chironomidae taxa from different waterbody types. Only taxa with significant values were shown (Significance codes: 0.001 ‘**’ 0.01 ‘*’); WBT 1—large rivers with fine substrate (silt, clay mud, and sand); WBT 4—small mountainous rivers and streams; WBT 5—slow flowing/stagnant waters (artificial canals and reservoirs).

WBT 1	A	B	Stat	*p* Value
*Polypedilum* gr. *scalaenum*	0.9536	0.5333	0.713	0.003 **
*Microchironomus tener*	1.0000	0.3333	0.577	0.010 **
*Dicrotendipes nervosus*	0.7904	0.4000	0.562	0.020 *
*Chironomus acutiventris*	1.0000	0.2000	0.447	0.043 *
*Parachironomus* gr. *gracilior*	1.0000	0.2000	0.447	0.050 *
*Paralauterborniella nigrohalteralis*	1.0000	0.2000	0.447	0.046 *
WBT 4				
*Tvetenia calvescens* agg.	0.8754	0.5000	0.662	0.010 **
*Rheotanytarsus* sp.	0.9977	0.3333	0.577	0.010 **
*Thienemanniella* sp.	0.9905	0.3333	0.575	0.020 *
*Micropsectra* sp.	0.9784	0.3333	0.571	0.006 **
*Micropsectra bidentata*	0.9749	0.3333	0.570	0.014 *
*Odontomesa fulva*	0.9699	0.3333	0.569	0.010 **
*Rheocricotopus effusus*	0.9577	0.3333	0.565	0.011 *
*Rheocricotopus chalybeatus*	0.8641	0.3333	0.537	0.011 *
WBT 5				
*Parachironomus* gr. *gracilior*	0.9907	0.4545	0.671	0.004 **
*Ablabesmyia longistyla*	0.7314	0.3636	0.516	0.033 *
*Polypedilum nubifer*	1.0000	0.1818	0.426	0.037 *

**Table 3:** Two-way PERMANOVA: differences between groups of communities in case of altitude (ALT) range, different waterbody types (WBT) and their interaction (WBT × ALT) (Bray–Curtis similarity matrix, 999 permutation); df—degrees of freedom, SS—sum of squares, MS—mean square.

Source	df	SS	MS	Pseudo-F	*p* Value	Unique Perms
WBT	4	27,437	6859.3	1.7234	0.001	994
ALT	2	10,725	5362.5	1.3474	0.033	998
WBT × ALT	4	19,840	4960.1	1.2462	0.013	997

**Table 4:** PERMANOVA: significance of differences between different waterbody types (WBT 1–WBT 5) (Bray–Curtis similarity matrix, with 999 permutations); WBT 1—large rivers with fine substrate (silt, clay mud, and sand); WBT 2—mix of large and medium rivers with coarse substrate (gravel, stones, and rocks); WBT 3—small watercourses with coarse substrate; WBT 4—small mountainous rivers and streams and WBT 5—slow flowing/stagnant waters (artificial canals and reservoirs).

	WBT3	WBT 2	WBT 4	WBT 5	WBT 1
WBT 3		0.0460 *	0.0055 *	0.0767	0.0002 *
WBT 2	0.0460 *		0.0006 *	0.6706	0.0014 *
WBT 4	0.0055 *	0.0006 *		0.0150 *	0.0001 *
WBT 5	0.0767 *	0.6706	0.0150 *		0.0021 *
WBT 1	0.0002 *	0.0014 *	0.0001 *	0.0021 *	

* denotes statistically significant values.

**Table 5:** The BIO-ENV analysis shows the four best combinations of environmental parameters for the Chironomidae community data matrix with Spearman’s rank correlation (Rho) and *p* values; (T—water temperature, O_2_%—oxygen saturation, NH_4_^+^—concentration of ammonium ions, NO_3_^−^—nitrates).

Model	*p* Value	Rho	Best Combination of Environmental Parameters
1	0.01	0.224	O_2_%, conductivity, NH_4_^+^, NO_3_^−^
2	0.01	0.222	O_2_%, conductivity, pH, NH_4_^+^, NO_3_^−^
3	0.01	0.216	T, O_2_%, conductivity, NH_4_^+^, NO_3_^−^
4	0.01	0.215	O_2_%, conductivity, pH, NO_3_^−^

## Data Availability

The data presented in this study are available on request from the corresponding author.

## Supplementary Materials

The following supporting information can be downloaded at: https://www.mdpi.com/article/10.3390/insects16050501/s1, Table S1: The list of sampling sites and their characteristics; Table S2: List of recorded taxa, their frequencies and occurrence in different water body types and altitudes; Table S3: Variability of physical and chemical parameters of different waterbody types and altitude.
