# Supplementary material for: Environmental Factors Determining the Distribution Pattern of Chironomidae in Different Types of Freshwater Habitats
Source: Insects. 2025 May 7;16(5):501. doi: 10.3390/insects16050501 (PMC12112228; doi:10.3390/insects16050501)
Supplement: Supplementary file 1 [file insects-16-00501-s001.zip › Supplementary Table S1.pdf]

Supplementary Materials

Table S1: The list of sampling sites and their characteristics

Water body type (WBT) codes: 1—large rivers with fine substrate (silt, clay mud, and sand); 2—mix of large and medium rivers with coarser substrate (gravel, stones, and rocks); 3—small watercourses with coarse substrate; 4—small mountain rivers and streams, 5—slow flowing/stagnant waters (artificial canals and reservoirs). The altitude codes: 1—localities up to 500 m a.s.l.; 2—localities from 500 to 1000 m a.s.l.; 3—localities above 1000 m a.s.l. Current velocity 1—slow, 2 —medium, 3 —fast

Sampling site	Altitude	Altitude code	WBT code	Current velocity
Tisovica	1167	3	3	2
Moravica	376	1	3	2
Kamenica	203	1	3	1
Peštan	127	1	3	2
Z. Morava	274	1	2	2
Katušnica	896	3	4	3
DTD Melenci	75	1	5	1
Grza	210	1	3	3
Ravanica 1	176	1	3	3
Ravanica 2	176	1	3	2
Tamiš	72	1	1	2
Štira	526	1	3	3
Krupinska reka	313	1	3	3
Drina	360	1	2	2
Nišava 1	564	2	2	3
Rasina	152	1	3	1
DTD N. Bečej	74	1	5	1
Tisa 1	101	1	5	1
Pek 1	402	1	2	3
Pek 2	110	1	2	3
Borkovac	117	1	5	1
Kudoš	94	1	5	1
Resava	123	1	3	2
Sava 1	80	1	1	2
Jablanica 1	481	2	3	1
Jablanica 2	422	1	3	1
Vučjanska reka 1	265	1	3	1
Vučjanska reka 2	611	2	3	1
Pusta reka	269	1	3	2
Toplica 1	217	1	3	2
Toplica 2	425	1	3	3
Južna Morava	228	1	2	1

Sampling site	Altitude	Altitude code	WBT code	Current velocity
Vlasina 1	329	1	3	2
Vlasina 2	360	1	3	3
Gradska reka	1309	3	4	3
Vlasina 3	846	3	4	2
Cvetkova reka	1350	3	4	2
Vrla 1	468	2	3	3
Vrla2	380	1	3	2
Nišava	378	2	2	2
Dunav 1	92	1	1	1
Begej	73	1	1	1
DTD Sombor	83	1	5	1
Svetićevo	97	1	5	1
Đetinja 1	458	1	3	3
Marića reka	948	3	3	1
Sjeničko jezero 1	1190	3	5	2
Sjeničko jezero 2	1062	3	5	2
Uvac 1	1109	3	5	2
Zlatarsko jezero 1	948	3	5	2
Uvac 2	948	3	3	1
Zlošnica 2	1106	3	3	1
Zlošnica 3	1106	3	3	1
Tisovica 2	1167	3	3	1
Tisovica 3	1167	3	3	1
Vapa 1	1066	3	4	1
Vapa 2	1023	3	4	1
Uvac 3	1066	3	3	1
Radoinja 1	948	3	3	1
Radoinja 2	948	3	3	1
Veljušnica reka 1	1190	3	3	1
Kladnica	1136	3	3	1
Dunav 2	91	1	1	2
Dunav 3	100	1	1	2
Tisa 2	75	1	1	2
Tisa 3	72	1	1	2
Sava 2	83	1	1	2
Sava 3	80	1	1	2
Dunav 4	70	1	1	2
V. Morava 1	132	1	1	2
Morava 2	69	1	1	2
Dunav 5	66	1	1	2

Sampling site	Altitude	Altitude code	WBT code	Current velocity
Dunav 6	52	1	1	2
Timok	45	1	2	2
