# Supplementary material for: Environmental Factors Determining the Distribution Pattern of Chironomidae in Different Types of Freshwater Habitats
Source: Insects. 2025 May 7;16(5):501. doi: 10.3390/insects16050501 (PMC12112228; doi:10.3390/insects16050501)
Supplement: Supplementary file 1 [file insects-16-00501-s001.zip › Supplementary Table S2.pdf]

Supplementary Materials

Table S2: List of recorded taxa, frequencies and occurrence in different water body types and altitudes.

**Taxa**	**F**	**WBT 1**	**WBT 2**	**WBT 3**	**WBT 4**	**WBT 5**	**ALT 1**	**ALT 2**	**ALT 3**
Ablabesmyia longistyla Fittkau 1962	0.11	*		*		*	*	*	*
Ablabesmyia monilis agg.	0.01			*				*	
Apsectrotanypus trifascipennis (Zetterstedt 1838)	0.04			*					*
Brillia bifida (Kieffer 1909)	0.03			*			*		
Brillia flavifrons (Johannsen 1905)	0.04			*	*		*	*	*
Chironomus acutiventris Wuelker Ryser & Scholl 1983	0.04	*					*		
Chironomus bernensis Kloetzli 1973	0.04		*	*	*		*		*
Chironomus commutatus Keyl 1960	0.01	*					*		
Chironomus riparius agg.	0.05	*		*		*	*		
Chironomus plumosus agg.	0.05	*		*		*	*		
Chironomus spp.	0.2	*	*	*		*	*		*
Cladopelma gr. *viridulum*	0.03	*		*			*		*
Cladotanytarsus sp.	0.16	*		*	*		*		*
Conchapelopia agg.	0.24		*	*	*		*	*	*
Corynoneura gr. *coronata*	0.04		*		*	*	*		*
Corynoneura lobata Edwards 1924	0.08			*	*			*	*
Corynoneura sp.	0.03			*			*		
Cricotopus bicinctus (Meigen 1818)	0.22	*	*	*	*	*	*	*	*
Cricotopus intersectus agg.	0.03	*	*				*		
Cricotopus gr. *sylvestris* sensu Hirvenoja 1973	0.19	*		*		*	*	*	*
Cricotopus gr. *tremulus*	0.11		*	*	*		*	*	*
Cricotopus triannulatus agg. sensu Moller Pillot 1984	0.04			*	*		*	*	*
Cricotopus trifascia Edwards 1929	0.01		*				*		
Cricotopus sp.	0.08		*	*	*	*	*	*	*
Cryptochironomus defectus (Kieffer 1913)	0.04	*				*	*		
Cryptochironomus denticulatus (Goetghebuer 1921)	0.01	*					*		
Cryptochironomus sp.	0.18	*	*	*		*	*	*	*
Demicryptochironomus vulneratus (Zetterstedt 1838)	0.03	*		*			*		*
Diamesa sp.	0.04			*	*		*		*
Dicrotendipes nervosus (Staeger 1839)	0.11	*		*		*	*		*
Dicrotendipes notatus (Meigen 1818)	0.04			*		*			*
Dicrotendipes lobiger (Kieffer 1921)	0.01	*					*		
Endochironomus gr. *dispar*	0.01			*			*		
Endochironomus albipennis Meigen 1830	0.03			*					*
Epoicocladius flavens Malloch 1915	0.07			*	*			*	*

Taxa	F	WBT 1	WBT 2	WBT 3	WBT 4	WBT 5	ALT 1	ALT 2	ALT 3
Eukiefferiella brevicalcar (Kieffer 1911)	0.01				*				*
Eukiefferiella claripenis (Kieffer 1911)	0.01			*			*		
Eukiefferiella sp.	0.05		*	*			*		*
Fleuria lacustris Kieffer 1924	0.01	*					*		
Gymnometriocnemus sp. A	0.01			*					*
Glyptotendipes gr. *barbipes*	0.03	*				*	*		
Glyptotendipes pallens agg.	0.03	*				*	*		
Glyptotendipes sp.	0.03	*				*	*		
Kloosia pulsilla (Linnaeus 1767)	0.01	*					*		
Harnischia fuscimanus (Kieffer 1921)	0.05	*		*			*		
Heleniella sp.	0.01			*					*
Heterotrissocladius gr. *marcidus*	0.01			*					*
Limnophyes sp.	0.01			*			*		
Macropelopia nebulosa (Meigen 1804)	0.03			*	*			*	
Microchironomus tener (Kieffer 1918)	0.07	*					*		
Micropsectra bidentata Goetghebuer 1921	0.05		*	*	*		*	*	*
Micropsectra sp.	0.04			*	*		*	*	*
Microtendipes chloris agg.	0.01		*				*		
Microtendipes pedellus agg. sensu Moller Pillot (1984)	0.14		*	*			*	*	*
Microtendipes rydalensis (Edwards 1929)	0.04			*				*	*
Metriocnemus gr. *eurynotatus* (*hygropetricus* type)	0.01			*					*
Monodiamesa sp.	0.01		*				*		
Monopelopia tenuicalcar (Kieffer 1918)	0.05			*			*	*	*
Nanocladius gr. *dichromus*	0.04	*		*	*		*		*
Nanocladius rectinervis (Kieffer 1911)	0.03		*	*			*		
Nilotanypus dubius (Meigen 1804)	0.01				*				*
Odontomesa fulva (Kieffer 1919)	0.07		*	*	*		*	*	*
Orthocladius sp.	0.24		*	*	*		*	*	*
Orthocladius frigidus (Zetterstedt 1838)	0.01			*			*		
Parachironomus gr. *gracilior*	0.08	*				*	*		*
Parachironomus frequens (Johannsen 1905)	0.01	*					*		
Paracladius conversus (Walker 1856)	0.09		*	*	*		*	*	*
Paracladopelma camptolabis (Kieffer 1913)	0.03			*		*			*
Paracladopelma nigritulum (Goetghebuer 1942)	0.03			*			*	*	
Paracladopelma sp.	0.01			*				*	
Parakiefferiella sp.	0.01			*				*	
Paralauterborniella nigrohalteralis (Malloch 1915)	0.04	*					*		
Paratanytarsus sp.	0.04	*		*		*	*		

**Taxa**	**F**	**WBT 1**	**WBT 2**	**WBT 3**	**WBT 4**	**WBT 5**	**ALT 1**	**ALT 2**	**ALT 3**
Paratanytarsus dissimilis (Johannsen 1905)	0.05			*	*			*	*
Parametriocnemus stylatus (Spaerck 1923)	0.12		*	*	*		*		*
Paratendipes albimanus (Meigen 1919)	0.09	*	*	*			*	*	*
Paratrichocladius rufiventris (Meigen 1830)	0.03			*			*		
Paratrissocladius excerptus (Walker 1856)	0.09			*	*		*	*	*
Phaenopsectra sp.	0.01				*				*
Polypedilum bicrenatum Kieffer 1921	0.03	*				*	*	*	
Polypedilum convictum (Walker 1856)	0.09	*		*			*		*
Polypedilum cultellatum Goetghebuer 1931	0.01			*			*		
Polypedilum laetum (Meigen 1818)	0.15		*	*	*		*	*	*
Polypedilum nubeculosum (Meigen 1804)	0.22	*		*	*	*	*		*
Polypedilum nubifer (Skuse 1889)	0.03					*	*		
Polypedilum gr. *scalaenum*	0.16	*		*			*		*
Polypedilum tritum (Walker 1856)	0.01	*					*		
Polypedilum uncinatum agg.	0.07	*		*			*		*
Potthastia gr. *gaedii*	0.03	*		*			*		*
Potthastia gr. *longimanus*	0.09		*	*	*		*	*	*
Procladius sp.	0.34	*	*	*	*	*	*	*	*
Prodiamesa olivacea (Meigen 1818)	0.24	*	*	*	*		*	*	*
Psectrocladius gr. *limbatelus/sordidelus*	0.01					*		*	
Psectrotanypus varius (Fabricius 1787)	0.03			*		*	*		*
Rheocricotopus chalybeatus (Edwards 1929)	0.05	*		*	*		*	*	*
Rheocricotopus effusus (Walker 1856)	0.04			*	*			*	*
Rheocricotopus fuscipes (Kieffer 1909)	0.26	*	*	*	*	*	*	*	*
Rheotanytarsus sp.	0.05	*		*	*		*	*	*
Stictochironomus pictulus (Meigen 1830)	0.01			*			*		
Stictochironomus sp.	0.04	*		*			*	*	
Synorthocladius semivirens (Kieffer 1909)	0.11		*	*	*		*	*	*
Tanytarsus spp.	0.39	*	*	*	*	*	*	*	*
Thienemaniella majuscula (Edwards 1924)	0.09		*	*	*		*		*
Thienemanniella clavicornis (Kieffer, 1911)	0.01	*					*		
Thienemanniella sp.	0.09	*		*	*		*	*	*
Tvetenia calvescens agg.	0.14		*	*	*		*	*	*
Virgatanytarsus arduennensis (Goetghebuer, 1922)	0.01			*					*
Xenopelopia sp.	0.04			*					*
Zavrelimyia sp.	0.03			*				*	*
