# Supplementary material for: Environmental Factors Determining the Distribution Pattern of Chironomidae in Different Types of Freshwater Habitats
Source: Insects. 2025 May 7;16(5):501. doi: 10.3390/insects16050501 (PMC12112228; doi:10.3390/insects16050501)
Supplement: Supplementary file 1 [file insects-16-00501-s001.zip › Supplementary Table S3.pdf]

Supplementary Materials

Table S3: Variability of physical and chemical parameters of different waterbody types WBT 1—large rivers with fine substrate (silt, clay mud, and sand); WBT 2—mix of large and medium rivers with coarser substrate (gravel, stones, and rocks); WBT 3—small watercourses with coarse substrate; WBT 4—small mountain rivers and streams, WBT 5—slow flowing/stagnant waters (artificial canals and reservoirs); and altitude ALT 1—localities up to 500 m a.s.l.; ALT 2—localities from 500 to 1000 m a.s.l.; ALT 3—localities above 1000 m a.s.l.

min	**Conductivity (µS/cm)**	**NH_4^+(mg/l)**	**NO_3^- (mg/l)**	**T**	**O_2%**	**O_2 mg/l**	**pH**
WBT 1	216.80	0.0150	0.3230	10.30	56.90	4.53	7.30
WBT 2	296.00	0.0250	0.5980	15.60	0.50	0.04	7.11
WBT 3	85.30	0.0100	0.0025	4.60	27.90	2.80	6.25
WBT 4	72.70	0.0580	0.0400	5.40	66.00	6.50	8.00
WBT 5	205.00	0.0025	0.0025	23.90	71.00	5.60	7.38
max							
WBT 1	533.00	0.5630	1.1500	26.30	119.40	10.45	8.41
WBT 2	1730.00	0.6140	8.8500	32.10	119.50	10.34	8.94
WBT 3	1823.00	4.3600	3.8280	23.40	142.80	11.08	8.73
WBT 4	441.00	2.0510	1.6290	23.80	109.00	85.00	8.46
WBT 5	1541.00	28.9000	7.2500	28.40	170.40	13.43	9.05
mean							
WBT 1	374.41	0.1082	0.7917	18.49	95.82	8.57	7.85
WBT 2	644.51	0.1510	3.2227	24.04	83.75	6.47	8.24
WBT 3	436.24	0.4667	0.9318	15.55	92.11	8.35	7.56
WBT 4	250.28	0.5763	0.6213	12.13	87.90	20.39	8.26
WBT 5	666.80	2.8582	2.0907	25.78	99.61	8.08	7.99
SD							
WBT 1	78.87	0.1603	0.2090	5.05	17.59	1.65	0.37
WBT 2	489.14	0.1970	3.4080	4.96	37.84	3.13	0.65
WBT 3	306.81	0.7836	1.0929	4.83	28.81	2.30	0.67
WBT 4	151.79	0.7492	0.6079	6.98	18.12	31.67	0.16
WBT 5	501.49	8.6498	3.1598	1.33	29.01	2.30	0.42

min	**Conductivity (µS/cm)**	**NH_4^+(mg/l)**	**NO_3^- (mg/l)**	**T**	**O_2%**	**O_2 mg/l**	**pH**
ALT 1	96.60	0.0025	0.0025	11.20	0.50	0.04	6.63
ALT 2	85.30	0.0500	0.0025	8.00	66.00	7.09	6.86
ALT 3	72.70	0.0100	0.0400	4.60	37.00	2.80	6.25
max							
ALT 1	1823.00	28.9000	8.8500	32.10	170.40	13.43	9.05
ALT 2	463.10	2.0510	2.0020	23.80	129.00	10.65	8.52
ALT 3	457.00	0.8000	1.6290	23.40	142.80	85.00	8.73
mean							
ALT 1	549.02	0.9714	1.7199	20.70	89.56	7.96	7.73

ALT 2	281.55	0.4073	0.6506	16.00	103.95	8.96	7.96
ALT 3	310.98	0.1613	0.3874	12.51	96.58	12.58	7.99
SD							
ALT 1	389.84	4.1319	2.2173	4.99	28.59	2.37	0.58
ALT 2	129.03	0.6760	0.7874	5.52	17.74	1.04	0.58
ALT 3	112.46	0.2269	0.5134	6.25	25.10	18.80	0.67
